# Internal Pudendal Artery Injury Following An Open Book Pelvic Fracture: A Case Report

**DOI:** 10.5704/MOJ.2011.030

**Published:** 2020-11

**Authors:** A Elhence, N Gahlot, A Gupta, P Garg

**Affiliations:** 1 Department of Orthopaedics, All India Institute of Medical Sciences, Jodhpur, India; 2 Department of Radiology, All India Institute of Medical Sciences, Jodhpur, India

**Keywords:** “open book” injury, pubic diastasis, internal pudendal artery, pelvic haemorrhage

## Abstract

Arterial haemorrhage is a potentially life threatening complication in severe pelvic ring injuries such as “open book” fractures. These injuries mostly implicate the posterior branches of the internal iliac artery. However, we report an unusual case wherein the source of bleeding was identified to be the internal pudendal artery and its branches. Patient was a 27-year-old male who presented to the emergency following an alleged history of road traffic accident and was diagnosed as a case of pelvic fracture (Young and Burgess Antero-Posterior Compression II) with sacral fracture (Denis type 2) with suspected urethral injury. Computerised Tomography (CT) angiogram revealed contrast extravasation from the right internal pudendal artery. However, digital subtraction angiography (DSA) was normal indicating spontaneous closure of the arterial bleeder. Surgical stabilisation of the fracture was carried out and subsequently, patient was discharged. This report serves to highlight that although uncommon, internal pudendal artery can be injured in hemodynamically unstable “open book” pelvic fractures and hence, must be always ruled out.

## Introduction

Shock and haemorrhage continue to be a major cause of concern amongst patients of pelvic trauma with mortality rates reported as high as 50% in some cases^1^. Although arterial bleed accounts for only 10% of total pelvic haemorrhage, it is more frequently associated with hemodynamic instability than other causes^1^. Usually, the mode of injury and the fracture pattern dictate the nature of injury to the pelvic arterial system. “Open book” fractures are potentially lethal due to associated urogenital injuries and massive bleeding (which has been seen to mostly implicate the posterior branches of the internal iliac artery)^1,2,3^.

However, we are reporting an unusual case where the source of bleeding in an “open book” pelvic fracture was the internal pudendal artery (which arises from the anterior division of the internal iliac artery)

## Case Report

**Fig. 1:**
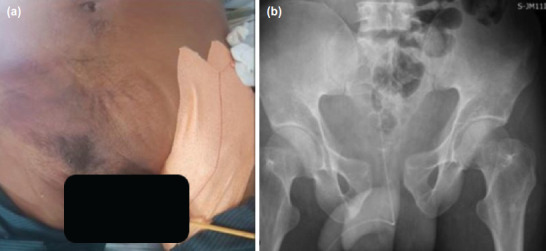
(a) Clinical image showing bruising involving right lower anterior abdominal wall with extension into scrotal sac. (b) Radiograph of pelvis reveals “open-book” pelvic injury with fracture of the left side of the sacrum.

Patient was a 27-year-old male who presented to the emergency with the chief complaint of sudden onset, severe lower abdominal pain and hematuria following an episode of road-traffic accident on the same day. Although conscious and oriented, he was tachycardic at the time of admission with hemodynamic parameters pointing towards hypovolemic shock. In accordance with advanced trauma life support (ATLS) protocols, patient’s vital parameters were first stabilised. Adequate resuscitation was commenced with oxygen support, intravenous fluids and packed red blood cells. Once hemodynamically stabilised, a detailed secondary survey was performed. The latter revealed guarding and tenderness in the lower half of the abdomen with significant bruising and discolouration of skin in the right iliac fossa extending towards the midline as well as a distinct scrotal swelling (Fig. 1a). Digital rectal examination (DRE) did not reveal a non-palpable prostate. Anal sphincter tone was normal. Systemic examination was negative for any head, chest or extremity injury.

**Fig. 2:**
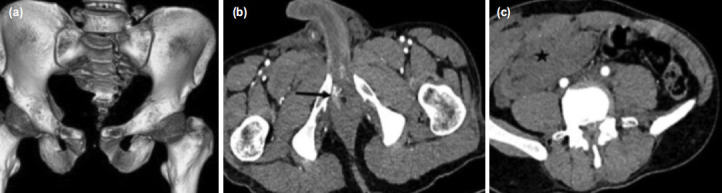
a) CT abdomen with pelvis, volume rendered image reveal fracture pattern identical to that seen on radiographs. (b) CT angiography reveal a small blob of contrast extravasation (black arrow) seen in right side of corpora cavernosa close to cavernosal branch of internal pudendal artery, (c) and hematoma (asterix) in right anterior abdominal wall and underlying part of peritoneal cavity.

**Fig. 3:**
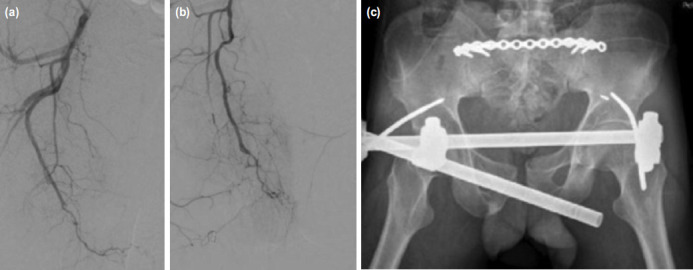
((a) Digital substraction angiography, selective right internal iliac artery (b) and superselective internal pudendal artery angiogram did not reveal any active contrast leak. (c) Post-operative radiograph of pelvis reveals posterior plating of the sacral fracture alongwith supra-acetabular fixator application for the pubic diastasis injury.

Focused assessment with sonography for trauma (FAST) carried out in the emergency room revealed free fluid in the pelvic cavity, likely haemoperitoneum. A complete blood count showed reduced haemoglobin levels (10.9g/dl). However, remaining blood investigations were all within normal limits. Patient was subsequently sent for radiographs (Fig. 1b) which showed an “open-book” injury to the pelvis (Young and Burgess Antero-Posterior Compression type II) with fracture of the sacrum (Denis type 2). Further evaluation with non-contrast computerised tomography (NCCT) of the pelvis with CT angiography of abdomen/pelvic cavity was also carried out. Volume rendering image (Fig. 2a) confirmed the fracture. On the other hand, CT angiogram revealed active contrast extravasation (Fig. 2b-black arrow) from cavernosal branch of right internal pudendal artery with pelvic hematoma (Fig. 2c-asterix) extending into the infra-umbilical abdominal wall, root of penis, corpora cavernosa and spongiosa of penis, explaining the skin discolouration and the scrotal-swelling. There were no findings to suggest intra/extra-peritoneal bladder rupture or urethral injury. It was decided to embolise the bleeder by digital subtraction angiography (DSA) by the intervention radiologist. However, selective right and left internal iliac artery (Fig. 3a) and superselective right internal pudendal artery (Fig. 3b) angiogram did not reveal any contrast extravasations or pseudoaneurysm formation indicating spontaneous closure of the bleeding artery. As a result, embolisation was not performed and patient remained hemodynamically stable after DSA.

Patient subsequently underwent tension band posterior plating of the sacral fracture along with closed reduction and supra-acetabular fixator application for the pubic diastasis injury (Fig. 3c). Post-operative check dressings were healthy looking. Patient was discharged on day four after surgery. He was prescribed pain medications, bed rest and gentle physiotherapy. He was also counselled with respect to the possibility of long-term impairment of his sexual functions and accordingly, advised rehabilitation and follow-up in the reproductive out-patient clinic. Scrotal swelling and skin discolouration resolved in about a week after surgery.

## Discussion

Pelvic ring injuries can be classified according to the Young and Burgess system of classification which is based primarily on the mechanism of injury. The fracture patterns described include- antero-posterior compression (APC), lateral compression (LC), vertical shear (VS) and combined mechanism (CM). However, this classification represents an anatomical description of pelvic ring injuries only. In contrast, the World Society of Emergency Surgery (WSES) classification takes into account both the Young and Burgess description of fracture patterns as well as the hemodynamic status of the patient and hence gives a better guide to overall patient management. There are four WSES grades (Grade1: APC/LC I and hemodynamically stable; Grade 2: APC/LC II-III and hemodynamically stable; Grade 3: VS/CM and hemodynamically stable and Grade 4: any hemodynamically unstable pelvic ring injury) which are further grouped under three classes of pelvic injuries (Minor: mechanically and hemodynamically stable/ WSES 1; Moderate: mechanically unstable but hemodynamically stable/ WSES 2/3; Severe: hemodynamically unstable/ WSES 4). Our patient was WSES Grade 4. Amongst these, fractures with severe ligament injury, namely APC II and III, VS and CM are associated with an increased risk of major vascular injury and subsequent hemodynamic instability. In such cases, the source of pelvic bleeding is usually threefold- the presacral venous plexus, fractured surfaces of the cancellous bone and from one or many branches of the internal iliac artery. Based on the sources of bleeding, various therapeutic options have been devised. These include pre-peritoneal pelvic packing, use of C- clamps or external fixators and embolisation of the injured arterial blood vessels^1^.

The latter has emerged as an important tool that directly helps in reducing mortality in patients of pelvic trauma. In a review of 24 studies that included 15,633 patients, Vaidya *et al*^1^ reported 74-100% success rate of therapeutic embolisation. However, the selection of vessels embolised was highly variable. According to the authors, vessels embolised in decreasing frequency were- internal iliac artery (67.2%), unnamed branches of the latter (17%), superior gluteal artery (4.4%), obturator artery (4.1%) and internal pudendal artery (3.2%).

The location of the arterial injury can usually be deciphered by the pelvic fracture pattern with the internal pudendal vessels most commonly implicated in fractures of the ischiopubic rami^1,4^. “Open book” injuries, on the other hand, tend to involve the superior gluteal arteries. This was validated in a study by O’Neill *et al*^2^ who found 35 cases of arterial injury amongst 39 patients of pelvic fracture. The authors noted that the internal pudendal artery was the most commonly injured blood vessel in patients of LC type injuries. The superior gluteal artery injury was more commonly seen in posterior pelvic ring fractures. Similarly, in a study by Metz *et al*^3^, 27 patients had LC type injury and 21 patients had APC type injury whereas one patient had VS pattern. Injuries to the arterial system were classified as internal iliac artery injury/anterior division injury/posterior division injury. In the LC group, there were 22 anterior division injuries and five posterior division injuries. In comparison, APC group had only five anterior division injuries and ten posterior division injuries.

In our study, the patient had had an active bleed from the cavernosal branch of the right internal pudendal artery which has put him at an increased risk for long term disability to his uro-genital functions. Such patients benefit from psychological counselling to address their concerns as well as regular follow-ups to monitor their progress.

To the best of our knowledge there have been very few cases in literature^4,5^ reporting the presence of anterior pelvic haemorrhage in patients of “open book” injuries. A summary of these cases and how they compare with ours is given in (Table I).

**Table I:** A summary of the incidence of anterior pelvic haemorrhage in open book fractures of the pelvis

S No.	Author/Year	Age/Sex	Fracture Pattern	Arterial Bleed
1	Wholey *et al*^5^ (1998)	45/F	Pubic symphysis and left iliopubic rami	Left internal pudendal artery
2	Margenthaler *et al*^4^ (2003)	13/M	Pubic symphysis, left sacroiliac joint and acetabulum	Anterior branches of internal iliac artery
3	Present study (2019)	27/M	Pubic symphysis with fracture left side of scarum	Right internal pudendal artery

In conclusion, internal pudendal artery injury must always be ruled out in a hemodynamically unstable “open-book” pelvic fracture. The key strategy involves initial hemodynamic stabilisation of the patient followed by appropriate use of computerised tomography as well as angiography to correctly identify the injured blood vessel. This will determine the outcome of a successful arterial embolisation and hence, overall patient outcome.
